# Maternal Omega-3 Nutrition, Placental Transfer and Fetal Brain Development in Gestational Diabetes and Preeclampsia

**DOI:** 10.3390/nu11051107

**Published:** 2019-05-18

**Authors:** Prasad P. Devarshi, Ryan W. Grant, Chioma J. Ikonte, Susan Hazels Mitmesser

**Affiliations:** Science and Technology, Pharmavite, LLC, West Hills, CA 91304, USA; rgrant@pharmavite.net (R.W.G.); cikonte@pharmavite.net (C.J.I.); smitmesser@pharmavite.net (S.H.M.)

**Keywords:** DHA, gestational diabetes, preeclampsia, placental transport

## Abstract

Omega-3 fatty acids, particularly docosahexaenoic fatty acid (DHA), are widely recognized to impact fetal and infant neurodevelopment. The impact of DHA on brain development, and its inefficient synthesis from the essential alpha-linolenic acid (ALA), has led to recommended DHA intakes of 250–375 mg eicosapentaenoic acid + DHA/day for pregnant and lactating women by the Dietary Guidelines for Americans. Despite these recommendations, the intake of omega-3s in women of child-bearing age in the US remains very low. The low maternal status of DHA prior to pregnancy could impair fetal neurodevelopment. This review focuses on maternal omega-3 status in conditions of gestational diabetes mellitus (GDM) and preeclampsia, and the subsequent impact on placental transfer and cord blood concentration of omega-3s. Both GDM and preeclampsia are associated with altered maternal omega-3 status, altered placental omega-3 metabolism, reduced cord blood omega-3 levels and have an impact on neurodevelopment in the infant and on brain health later in life. These findings indicate lower DHA exposure of the developing baby may be driven by lower placental transfer in both conditions. Thus, determining approaches which facilitate increased delivery of DHA during pregnancy and early development might positively impact brain development in infants born to mothers with these diseases.

## 1. Introduction

Eicosapentaenoic acid (EPA, C20:5) and docosahexaenoic acid (DHA, C22:6) are omega-3 polyunsaturated fatty acids that are well-studied in humans [1,2,3]. Because they can be synthesized in the body from their precursor, alpha-linolenic acid (ALA, C18:3), EPA and DHA are not considered essential fatty acids. However, inefficient conversion of ALA to EPA and DHA [4,5] has led to recommendations to include food and dietary supplements as sources of EPA and DHA.

Wide recognition of the importance of EPA and DHA in infant development has led to recommendations for pregnant and lactating women from many regulatory bodies [6,7,8,9,10] and medical/scientific societies [11,12,13,14,15,16,17]. In the US, the American Academy of Pediatrics, American College of Obstetricians and Gynecologists, and March of Dimes recommend an intake of fish or omega-3 fatty acids resulting in ~200 mg DHA/day, and the Dietary Guidelines for Americans (DGA) recommends ~250–375 mg EPA + DHA/day. Despite these recommendations to promote the intake of omega-3 fatty acids, consumption of omega-3 fatty acids remains low across the US population [18], specifically in child-bearing-age, pregnant and lactating women [19]. Up to 95% of child-bearing-age and pregnant women do not meet the DGA recommendations for EPA + DHA intake [19]. Because of the impact DHA has on fetal development, the gap between the recommended and the actual DHA intake is concerning.

It is well established that DHA is an important structural component of the human brain and retina [20]. In the retina, DHA represents ~80% of all polyunsaturated fatty acids, while 60% of the dry weight of the brain is fatty acids, of which DHA is the major omega-3 fatty acid [21,22]. Omega-3 fatty acid deficiency leads to low levels of DHA in the cerebral cortex of the offspring and affects learning ability in animal models [23,24]. During fetal and infant development, membranes of the retina and brain grey matter become enriched with DHA [20]. During the third trimester, DHA selectively accumulates in the brain at a higher rate than other fatty acids [25]. This incorporation of DHA continues at these high rates until age 2 [26]. The first two years of life are an essential period of neurodevelopment for infants [27]. In the brain, DHA plays many important roles, including cell signaling, regulation of gene expression and neurotransmission [28,29,30]. In 60-day old rats, a diet deficient in omega-3 fatty acids led to altered physiology of neurotransmitters such as serotonin and dopamine, which are important for brain function [31]. Additionally, DHA deficiency led to decreased neuron size in various areas of the brain such as the hypothalamus and the hippocampus in animal models [32]. Normal neuron size is essential for their function in the brain [32]. Moreover, it was shown that maternal omega-3 deficiency not only affects neuronal size, but also neurogenesis in animals [33]. A lower rate of neurogenesis may affect cognitive function later in life [33]. Human studies have demonstrated that maternal omega-3 fatty acid supplementation increases maternal as well as fetal levels of DHA. DHA supplementation (200 mg/day) starting at the 21st week of gestation through lactation showed an increase in maternal red blood cells (RBCs) DHA at 37 weeks gestation and 3 months post birth. Additionally, this led to increased DHA in human milk and RBC DHA in the infant [34]. The benefits of omega-3 fatty acid supplementation during pregnancy have been well documented in the infant. After 1500 mg/week supplementation of DHA (as a functional food) during pregnancy, 9-month-old infants had improved performance on problem-solving tasks compared to the non-supplemented group [35]. Fish oil supplementation (2200 mg DHA and 1100 mg EPA per day) starting at 20 weeks gestation until birth showed an improvement in hand and eye coordination in the children at two and half years of age [36]. Additionally, hand and eye coordination scores positively correlated with omega-3 fatty acid levels in cord blood RBCs [36]. Supplementation of 600 mg/day of DHA from 14.5 weeks of gestation until birth, led to high levels of sustained attention in infants at 4, 6 and 9 month of age, while sustained attention declined in the control group during this period [37], suggesting the importance of omega-3 fatty acids in fetal brain development and function.

Despite the importance of omega-3 fatty acid intake on fetal brain development [38], most pregnant and child-bearing age women in the US are consuming less than the recommended amount [19]. While it is known that socioeconomic status influences infant cognitive development and nutrition [39,40], this review focuses on the effect of low placental omega-3 transfer in gestational diabetes mellitus (GDM) and preeclampsia on neurodevelopment. In addition to the low omega-3 fatty acid intake among pregnant women, in women with GDM and preeclampsia the transfer of DHA through the placenta to the fetus is compromised [41,42]. GDM is defined as impaired glucose tolerance first detected during pregnancy and is associated with various implications for fetal growth [43,44]. Preeclampsia is defined as systolic blood pressure of ≥140 mm Hg or diastolic blood pressure ≥90 mm Hg on two occasions at least 4 h apart, along with proteinuria, thrombocytopenia, renal insufficiency, impaired liver function, pulmonary edema, or cerebral or visual symptoms after 20 weeks of gestation [45]. Both GDM and preeclampsia are pregnancy disorders that have effects on fetal growth and development [43,44,46]. Due to the importance of omega-3 fatty acids in fetal brain development, it is important to understand the underlying mechanisms likely responsible for the reduced transfer of DHA to the fetus in GDM and preeclampsia. Here, we review the association of GDM and preeclampsia with maternal omega-3 fatty acid status, the impact of these disorders on the transfer of omega-3 fatty acids to the fetus, and subsequent effects on fetal brain development.

## 2. Gestational Diabetes

### 2.1. Maternal and Fetal Effects

In the course of gestation in all pregnancies, including normal pregnancies, insulin resistance increases progressively and is highest in the third trimester [47]. In normal pregnancies, the pancreatic beta cells produce higher levels of insulin which prevents hyperglycemia, whereas, in GDM, the response of these beta cells is inadequate, leading to hyperglycemia [47]. GDM is defined as impaired glucose tolerance first detected during pregnancy and is associated with various implications for fetal growth [43,44]. According to the 2014 analysis by the Centers for Disease Control and Prevention, GDM affects 9.2% of all pregnancies in the US [48]. Mechanisms involving progesterone and glucocorticoids and their effect on insulin sensitivity have been suggested to be responsible for the increase of insulin resistance during gestation [49,50]. Progesterone secretion increases during pregnancy and it has been shown to decrease the ability of insulin to suppress synthesis of glucose in the liver [49]. Cortisol is a glucocorticoid that increases during pregnancy and has have been shown to affect skeletal muscle insulin signaling [50,51]. Glucocorticoids reduce phosphorylation of the insulin receptor and reduce the content of insulin receptor substrate-1, which is involved in insulin signaling, in the skeletal muscle [50]. Women with GDM have higher levels of insulin resistance compared to normal pregnancy controls [52]. The combination of elevated insulin resistance and inadequate beta cells response both likely contribute to hyperglycemia in GDM [47,52,53]. GDM has a complicated etiology involving numerous metabolic and genetic factors with many underlying mechanisms [54]. A large prospective cohort study found that aging, obesity, non-white ethnicity, family history of diabetes and smoking increases the risk for developing GDM [55]. In addition, the development of GDM during pregnancy increases the risk of GDM in future pregnancies [56].The etiology of GDM is complex and comprises lifestyle and genetic risk factors, with many potential mechanisms.

GDM leads to adverse long-term effects including a higher risk of developing metabolic diseases in the mother and the child [57,58]. In addition to these adverse effects, the presence of diabetes during pregnancy is associated with impaired neurodevelopment [59,60,61,62,63,64]. Infants of mothers with GDM showed lower mental and psychomotor development indexes compared to infants of non-GDM mothers at 6 months of age [59]. Similarly, children below 9 years of age, of mothers with GDM, had lower verbal IQ scores than children of non-GDM mothers [60]. These children also scored low compared to children of non-GDM mothers in the Pollack tapper test, which is used to detect learning disabilities [60]. GDM also affects language development during childhood [61]. Children (18 and 30 months) of GDM mothers had lower expressive vocabulary and grammar scores compared to children of non-GDM mothers [61]. These children were at a 2.2-fold higher risk of having impaired language development [61]. In children of GDM mothers at 8 years of age, a trend towards lower IQ scores compared to children of non-GDM mothers was seen [64]. As DHA is an important nutrient for fetal brain development [38], it has been suggested that the effects on neurodevelopment may be as a result of low cord blood levels of DHA seen in GDM [59]. Additionally, in animal models, it has been suggested that inflammation due to GDM can lead to adverse effects on neurodevelopment in the offspring [65]. In totality, these studies demonstrate the influence of GDM on neurodevelopment.

### 2.2. Placental Transfer of Omega-3 Fatty Acids in GDM

The negative neurodevelopment effects observed in children of mothers with GDM could be a result of lower omega-3 fatty acid transfer from the mother to the fetus [66]. Maternal DHA is the primary source of DHA for the fetus as it cannot be synthesized in the fetus or the placenta [41]. The placenta preferentially absorbs DHA for transfer to the fetus during pregnancy and this is demonstrated by higher cord vein DHA levels compared to maternal blood DHA levels [67]. In GDM pregnancies, cord vein blood levels of DHA are lower than the levels in normal pregnancies [59,66,68]. Specifically, maternal DHA levels have been shown to be 11–44% higher in GDM, while cord blood levels were lower compared to normal pregnancies, indicating a possibility of reduced placental transfer of DHA to the fetus in GDM [69]. Thomas et al. similarly found that cord vein DHA was lower in GDM versus normal pregnancy [68]. Wijendran et al. observed that GDM mothers had a higher intake of omega-3 fatty acids during the third trimester of pregnancy compared to normal pregnancy controls [66]. Due to higher dietary intake, GDM mothers also had significantly higher maternal DHA erythrocyte content [66]. However, in spite of higher DHA intake and maternal DHA erythrocyte levels, DHA in cord vein erythrocytes was significantly lower (37% lower) than in normal pregnancy controls [66]. In this study, maternal plasma DHA positively correlated with DHA in fetal erythrocytes in the normal pregnancy control group, however this correlation was not present in GDM mothers [66]. Moreover, maternal hemoglobin A1c (HbA1C) levels were inversely related to fetal erythrocyte DHA content in GDM mothers, which suggests a relation between GDM and transfer of DHA to the fetus [66].

Several mechanisms have been proposed for the reduction in placental transfer of DHA in GDM. Peroxisome proliferator-activated receptor (PPAR)-alpha and PPAR-gamma belong to the family of nuclear receptors that control various fatty acid metabolism related genes, including genes related to fatty acid transport [70]. PPAR-alpha mainly controls fatty acid oxidation related genes and PPAR-gamma controls adipogenesis related genes [70]. PPAR-alpha was downregulated in GDM placentae compared to uncomplicated term placentae [71]. Also, PPAR-gamma had lower gene and protein expression in GDM placenta [71]. Lipoprotein lipase (LPL), which is expressed in the microvillous plasma membrane of the placenta, is needed to release free fatty acids from triglycerides in circulating lipoproteins, leading to uptake by the placenta [72]. In pregnancies complicated by type-1 diabetes, LPL activity in the microvillous plasma membrane of the placenta was increased by 39% compared to the normal pregnancy control, but no difference was seen in GDM pregnancies indicating that LPL is not involved in mechanisms related to reduced placental transfer of DHA in GDM [72,73]. The transport of fatty acid through the placenta is orchestrated by a number of proteins, including: Fatty acid transfer proteins such as fatty acid transport protein (FATP)-1-6, cluster of differentiation 36 (CD-36), fatty-acid-binding protein (FABP)-pm, placental-FABP-pm in the plasma membrane and FABP-1, 3, 4, 5, 7 [74]. In GDM placenta, it was seen that expression of FATP-1 and FATP-4 were decreased, while expression of CD-36 and FATP-6 were increased compared to normal pregnancy controls [75]. The major facilitator superfamily domain-containing protein 2A (MFSD2A) was identified as a brain choline and DHA transporter [76]. It was seen that MFSD2A was significantly reduced in GDM placentae [77]. DHA percentage in the cord vein blood correlated with levels of MFSD2A, suggesting a possible role in placental transfer of DHA to the fetus [77]. These alterations could be possibly responsible for reduced DHA transfer across the placenta.

Some studies have investigated the effect of omega-3 fatty acid supplementation during pregnancy on GDM [78,79,80]. Zhou et al. investigated the effect of DHA supplementation on the incidence of GDM [81]. DHA intake of 800 mg/day did not affect risk of developing GDM [81]. In GDM women, 800 mg/day of DHA supplementation starting at 17–33 weeks of gestation, increased DHA in maternal plasma and RBCs, but failed to increase DHA in cord vein plasma and RBCs. More studies investigating the effects of early DHA supplementation on placental expression of fatty acid metabolism-related genes and fetal outcomes are needed.

## 3. Preeclampsia

### 3.1. Maternal and Fetal Effects

Preeclampsia occurs in ~3% of pregnancies and negatively impacts both maternal and fetal health outcomes [45]. The American College of Obstetricians and Gynecologists defines preeclampsia as a systolic blood pressure of ≥140 mm Hg or diastolic blood pressure ≥90 mm Hg on two occasions at least 4 h apart, which co-occurs with proteinuria, thrombocytopenia, renal insufficiency, impaired liver function, pulmonary edema, or cerebral or visual symptoms after 20 weeks of gestation [45]. Although preeclampsia typically develops during the second trimester of pregnancy, its disease pathogenesis likely begins in the first trimester during implantation. Delivery of nutrients from the mother to the fetus depends on placental vasculature. Placental vascular development requires remodeling of the uterine spiral arteries from narrow vessels to wider, low resistance vessels [82]. Impairment of vascular remodeling during early gestation is thought to result in the later development of preeclampsia. Proposed mechanisms leading to preeclampsia include impairment of endothelial function and a maternal immune response to the invading trophoblast cells that remodel the spiral arteries [82]. During pregnancy, the maternal immune system develops tolerance to cells of the fetus [83]. Defects in the development of tolerance and activation of a pro-inflammatory response may be contributing factors to the development of preeclampsia [83].

Impairment of placental vascularization during preeclampsia limits efficient delivery of nutrients and oxygen to the fetus. Low oxygen levels lead to increased rates of neuronal apoptosis in experimental models of preeclampsia and perinatal asphyxia [84]. In addition to the sensitivity of neurons to low oxygen levels, hypoxia can drive tissue inflammatory responses. Preeclampsia upregulates placental expression of proinflammatory cytokines (tumor necrosis factor-α, Interleukin-1 (IL-1), and IL-18) and downregulates the anti-inflammatory cytokine IL-10 [85]. Indeed, human term placental explants exposed to low oxygen levels had increased production of tumor necrosis factor-α, IL-1α and β [86]. In ischemic brain injury, there is upregulation of both IL-1β and IL-18. Mice that lack production of IL-18 or were treated with an intracerebroventricular injection of an IL-1 receptor antagonist have reduced ischemic brain injury [87,88]. Hypoxia additionally promotes the production of reactive oxygen species and the development of oxidative stress, which can also contribute to neuronal apoptosis [89]. The impact of hypoxia on neuronal apoptosis, inflammation and oxidative stress likely function together to negatively impact brain development. In children, prenatal hypoxia is a known contributor to impaired cognitive development [90]. Specifically, preeclampsia negatively impacts several aspects of cognitive function including verbal reasoning and executive function during early childhood [91]. Preeclampsia may also impact brain health later in life. Children from mothers with preeclampsia had higher depression symptoms during adulthood [91]. These findings suggest that preeclampsia may impact the brain across the lifespan. To establish a link between preeclampsia and brain function, some studies have analyzed brain structure. Initial studies have demonstrated that children from preeclamptic pregnancies exhibit differences in the structure and connectivity of limbic system components, an area of the brain linked with mood, behavior and cognition [91]. Although the underlying factors that drive preeclampsia are not completely understood, evidence indicates that preeclampsia impacts maternal and fetal health, as well as long-term development.

### 3.2. Placental Transfer of Omega-3 Fatty Acids in Preeclampsia

Omega-3 fatty acids, and particularly DHA, are critical to the structure and function of developing nervous system cells. Several factors influence the availability of DHA and EPA to fetal tissues, including: Dietary intake, endogenous synthesis from alpha-linolenic acid, transport within the blood for delivery to placental tissues, placental tissue uptake and transfer to cord blood. Several case-controlled studies have demonstrated that during the third trimester or postpartum, blood levels of omega-3 fatty acids are reduced in mothers with preeclampsia compared to normal pregnancy. An initial study of postpartum mothers indicated that women in the lowest tertile of erythrocyte omega-3 fatty acid content were 7.6 times more likely to have preeclampsia compared with those in the highest tertile [92]. Kulkarni et al. found that at delivery, plasma DHA levels were reduced in preeclamptic vs. normotensive pregnancy, and this difference was also reflected in infant cord blood samples [93]. An additional study found that third trimester levels of omega-3 fatty acids were reduced in mothers with preeclampsia or intrauterine growth restriction (IUGR) compared to normal pregnancy [94]. Cord blood taken from these subjects also indicates a reduction in total omega-3 fatty acids and DHA compared to normal pregnancy [94]. Although differences in maternal omega-3 fatty acids during the third trimester are apparent, they don’t account for exposure across the duration of pregnancy. A more recent study examined longitudinal changes of serum fatty acid proportions in normotensive and preeclamptic pregnant women. This study demonstrated reduced circulation of total omega-3 fatty acids, DHA and arachidonic acid at 16–20 weeks but not at 26–30 weeks or at delivery in preeclamptic pregnant women [95]. This study did confirm lower cord blood levels of both DHA and total omega-3 fatty acids at delivery with preeclampsia, which were similar to the differences found by Kulkarni et al. [95]. This study also revealed significant correlation of maternal plasma DHA with cord plasma DHA across all three time points [95], highlighting reduced maternal omega-3 fatty acids during preeclampsia.

Given that maternal blood levels of omega-3 fatty acids are low during preeclampsia, a key question is whether the placenta can compensate by upregulating the trafficking, synthesis and/or transfer of omega-3 fatty acids. The fatty acid composition of placentas from mothers with preeclampsia is different from those with normal pregnancies. Wang et al. demonstrated that placentas from preeclamptic women have reduced total non-esterified polyunsaturated fatty acids, including lower total omega-3 fatty acids and DHA compared to placenta from normal pregnant women [96]. The observation of reduced placental DHA has been confirmed by other studies [95,97,98]. An initial study comparing omega-3 fatty acid composition in different areas of the placenta indicates regional differences between normotensive controls, term pregnancy with preeclampsia and preterm pregnancy with preeclampsia. DHA and total omega-3 fatty acid concentrations were consistently lower in the central fetal and maternal areas of the placenta in mothers with preeclampsia and preterm delivery [97]. Additionally, in the central fetal area there were higher concentrations of ALA with preterm delivery and preeclampsia, indicating a possibility of inefficient conversion of ALA to DHA [97]. Because there were no matched normotensive preterm controls, it is unclear whether these changes are driven by gestational time or the severity of disease.

The placenta transports omega-3 fatty acids and expresses enzymes which contribute to the metabolism of ALA into EPA and DHA. Thus far, studies do not indicate increased capability for EPA + DHA synthesis or transport in preeclampsia. The expression of genes responsible for placental lipid transport (FATP 1 and 4) and fatty acid elongation (fatty acid desaturase 1) were reduced with preeclampsia, while others (fatty acid desaturase 2 and FABP-3) were not altered [95]. This indicates that during preeclampsia there may be a down regulation of the capacity to transport lipid in the placenta, and potentially a reduced capacity to synthesize DHA from ALA in placental tissue. Placental mRNA expression of MFSD2A, a DHA transporter in the brain, is reduced during severe preeclampsia compared to normotensive pregnancy [99]. However, it is unknown what the impact of this reduction is on fetal development. Collectively, these observations indicated altered transport and metabolism of omega-3 fatty acids during preeclampsia. These results are supported by reduced omega-3 fatty acids in placental tissue and cord blood from mothers with preeclampsia versus normal pregnancy. It is currently unknown whether cord blood DHA levels are responsive to increased dietary intake during preeclampsia.

A number of intervention trials were conducted to understand if supplemental omega-3 fatty acids from fish oil could impact maternal outcomes associated with preeclampsia. These studies have been largely ineffective [100] and vary widely in dose and composition of omega-3 fatty acids, as well as gestational age at the onset of the intervention [101]. Although studies have focused on the mother, this does not preclude impact on the baby. It was noted by Kulkarni et al. that cord blood DHA levels were correlated with serum DHA levels in mothers with preeclampsia. This raises the possibility that increasing maternal omega-3 fatty acid status may increase omega-3 fatty acid exposure of the developing baby. Future studies are needed to determine whether increased maternal prenatal omega-3 fatty acid intake can improve cord blood DHA and developmental outcomes in babies born to mothers with preeclampsia.

## 4. Conclusions

Despite the recommendations from various authorities, 95% of pregnant and child-bearing-age women do not consume enough omega-3 fatty acids [19,38]. Transfer of omega-3 fatty acids through the placenta to the cord blood and fetus is impaired in GDM and preeclampsia [41,42,75,77,95]. This is reflected in both conditions by lower cord vein DHA levels compared to normal pregnancies [66,93]. Given the role DHA plays in fetal neurodevelopment, lower DHA transfer to the fetus may contribute to impaired neurodevelopment.

Although it is apparent that GDM and preeclampsia are associated with lower cord blood levels of DHA, the impact of DHA and omega-3 fatty acid supplementation on fetal neurodevelopment in this population remains unclear. In GDM, increasing DHA intake later in pregnancy did not increase cord blood levels, possibly due to impaired DHA placental transfer [102]. Future studies in both GDM and preeclampsia need to focus on very early interventions. Such studies could elucidate the time course of serum fatty acids during pregnancy and the impact of DHA supplementation on maternal blood, placental and cord vein DHA levels. Additionally, there is a need to further link infant neurodevelopmental outcomes to omega-3 fatty acid intake. One factor that may influence the effects of omega-3 fatty acids on neurodevelopment may be socioeconomic status. Most of the studies discussed did not evaluate the impact of socioeconomic status on offspring neurodevelopment. This is a limitation, as socioeconomic status has been suggested to affect neurodevelopment and omega-3 nutrition [39,40]. Future studies are needed to determine the effect of socioeconomic status on maternal nutritional interventions and offspring neurodevelopment. While DHA intake prior to and during pregnancy is important, postnatal DHA intake could be an impactful opportunity to increase delivery of DHA to the breast-fed new born baby. Interventions supporting increased DHA consumption might positively impact neurodevelopmental outcomes associated with GDM and preeclampsia.
